# miR-21a-5p targets DDX58 to suppress TLR2/NF-κB signaling and protect the blood–brain barrier after injury

**DOI:** 10.1007/s11033-026-13010-3

**Published:** 2026-10-25

**Authors:** Jing Yang, Ning Tang, Ruanxian Dai, Hao Fu, Shiyi Li, Qiang Meng

**Affiliations:** 1 Department of Intensive Care Unit, Yan’an Hospital of Kunming City, Yan’an Hospital affiliated to Kunming Medical University, Kunming, 650051 China; 2 Department of Neurology, The First People’s Hospital of Yunnan Province, The Affiliated Hospital of Kunming University of Science and Technology, 157 Jinbi Rd, Xishan district, Kunming, 650032 Yunnan Province China; 3 Faculty of Life Science and Technology, Kunming University of Science and Technology, Kunming, 650500 China; 4 College of Science, Minzu University of China, Beijing, 10081 China

**Keywords:** Blood-brain barrier, miR-21a-5p, DDX58, TLR2/NF-κB pathway, Tight junction proteins, MMP-2/9

## Abstract

**Background:**

Damage to the blood-brain barrier (BBB) plays a pivotal role in the pathogenesis of various neurological disorders. miR-21a-5p has been implicated in the modulation of BBB injury. Understanding the molecular targets of miR-21a-5p is crucial for developing targeted interventions in BBB repair.

**Methods:**

The target gene of miR-21a-5p was identified through bioinformatics analysis and validated using a dual luciferase reporter assay. The expression of target gene was further confirmed in both in vivo and in vitro models of BBB injury. BBB injury cell models were employed to assess the effects of target gene overexpression and silencing on brain microvascular endothelial cell (BMEC) activity, as well as on the TLR2/NF-κB signaling pathway and associated BBB proteins. miR-21a-5p overexpression and silencing were utilized to assess the impact of target gene on BBB integrity.

**Results:**

DDX58 was identified as a direct target of miR-21a-5p and is shown to modulate BBB injury. DDX58 expression was found to be upregulated in models of BBB damage. Exosomes containing miR-21a-5p mitigated BBB damage, which was associated with downregulated DDX58 expression in vivo and in vitro. Overexpression of DDX58 in BMECs resulted in reduced cellular viability, increased expression of TLR2 and phosphorylated p65 (p-p65), as well as elevated levels of matrix metalloproteinases (MMP-2/9), which are associated with BBB disruption. This was accompanied by a reduction in tight junction proteins, including claudin-5, occludin, and ZO-1. Conversely, silencing DDX58 expression exhibited the opposite effects. Both miR-21a-5p treatment and DDX58 interference alleviated BBB injury. Co-overexpression of DDX58 during miR-21a-5p treatment reversed the protective effects of miR-21a-5p, exacerbating BBB injury.

**Conclusion:**

miR-21a-5p targets and downregulates DDX58, which is associated with reduced expression of TLR2/NF-κB-related markers, decreased MMP-2/9 levels, and protection of tight junction integrity.

**Supplementary Information:**

The online version contains supplementary material available at https://doi.org/10.1007/s11033-026-13010-3.

## Introduction

The blood-brain barrier (BBB) is a highly selective interface within the brain, composed of endothelial cells, pericytes, and glial cells¹, which functions as a crucial filter to protect the brain from harmful substances in the bloodstream and maintain homeostasis [1]. The endothelial cells that form the BBB’s structural and functional core are key to its selective permeability. Early markers of BBB dysfunction include the degradation of the tight junction glycocalyx on endothelial cells. These tight junctions play an essential role in maintaining BBB integrity, and their disruption is often an early sign of BBB breakdown [1, 2]. Endothelial cells also maintain interconnectivity through direct interactions with astrocytes (ACs), which facilitate the formation of large multiprotein complexes [3]. Among these, the transmembrane tight junction proteins claudin-5 and occludin, as well as the auxiliary protein ZO-1, which links to the actin cytoskeleton, are critical for maintaining the barrier’s integrity and selective filtration function [3]. Matrix metalloproteinases (MMPs), particularly MMP-2 and MMP-9, contribute to BBB damage by degrading tight junction proteins, exacerbating barrier disruption [3–5]. Moreover, inflammation-driven mechanisms of BBB injury are central to the progression of various conditions, including ischemic injury [6]. Both peripheral inflammatory mediators [7] and inflammation within brain endothelial cells contribute to BBB disruption, highlighting the role of inflammation and tight junction disruption as key risk factors for BBB damage [8]. Impaired BBB function is implicated in numerous diseases, such as aging [1], neuroimmunological disorders [9], and cerebrovascular diseases [10, 11]. Therefore, developing targeted strategies to repair and maintain BBB integrity is of critical importance.

MicroRNA-21a-5p (miR-21a-5p) is a multifunctional microRNA implicated in a variety of diseases, including traumatic spinal cord injury inflammation [12], periodontitis [13], acute lung injury [14], diabetes [15], and renal fibrosis [16]. Our previous research has demonstrated that exosomes derived from bone marrow mesenchymal stem cells (BMSC-exo) carrying miR-21a-5p can mitigate BBB disruption and hemorrhagic transformation [17]. However, the specific molecular targets through which miR-21a-5p exert protective effects on the BBB remain unclear. DDX58 encodes retinoic acid-inducible gene-I (RIG-I), a cytoplasmic pattern recognition receptor (PRR) that plays a pivotal role in immune responses by recognizing viral RNA [18]. DDX58 has been implicated in various diseases, including colorectal cancer [19], lupus nephritis [20], tumors [21], and hepatocellular carcinoma [22], though its role in BBB function has not been previously explored.

In this study, we employed bioinformatics analysis and luciferase assays to identify DDX58 as a direct target of miR-21a-5p. Our results indicate that miR-21a-5p modulates BBB injury by regulating the Toll-like receptor 2 (TLR2)/NF-κB signaling pathway through DDX58. These findings shed light on the molecular mechanisms underlying the miR-21a-5p/DDX58 pathway in BBB injury, providing valuable insights for future therapeutic strategies targeting DDX58 to mitigate BBB damage and facilitating the clinical translation of miR-21a-5p-related research.

## Methods

### Cell processing and luciferase assay

**Fig. 1:**
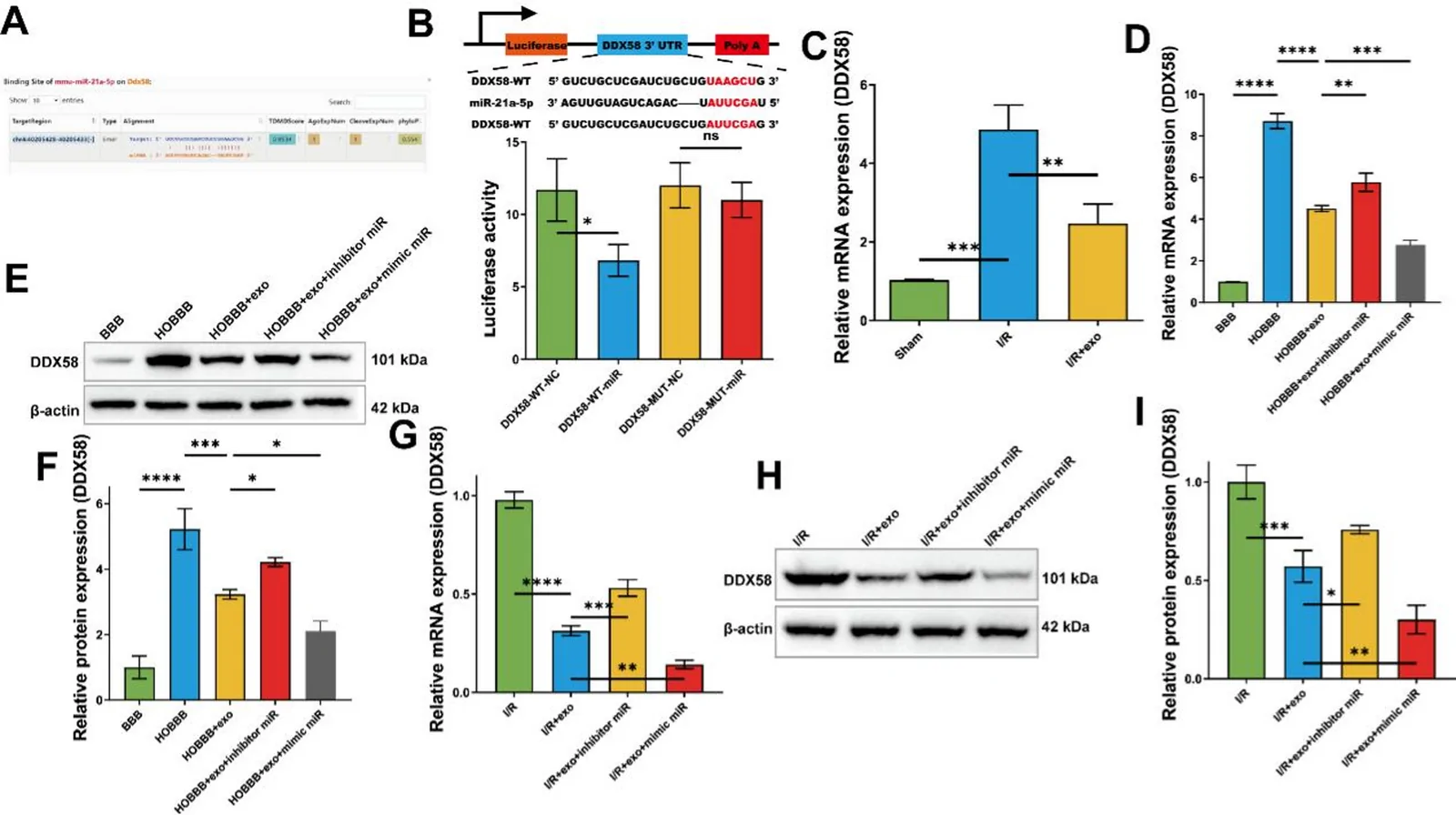
miR-21a-5p is associated with downregulated DDX58 expression in BBB damage. **A**: The target genes of miR-21a-5p were identified using bioinformatics analysis. **B**: The targeted binding between miR-21a-5p and DDX58 was detected using a dual luciferase assay. **C**: The mRNA expression of DDX58 in BBB-damaged and miR-21a-5p-treated in vivo models was detected by RT-qPCR. **D-F**: The mRNA (D) and protein (E-F) expression of DDX58 in BBB-damaged and miR-21a-5p differentially expressed in vitro cell models was detected by RT-qPCR and western blot. **G-I**: The mRNA (G) and protein (H-I) expression of DDX58 in the BBB-damaged and miR-21a-5p differentially expressed in vivo animal model was detected by RT-qPCR and western blot. ns indicates no significant difference between the two comparison groups; *p < 0.05; **p < 0.01; ***p < 0.001; ****p < 0.0001

Human renal embryonic kidney 293T cells were purchased from BeNa Culture Collection (BNCC353535, China). When 293T cells reached 90% confluence, they were digested with 0.25% trypsin (1894145, Gibco, USA) to collect cells, which were then seeded into 12-well plates at 2 × 10⁴ cells per well. Each group consisted of three replicate wells. Transfection was performed when cells reached approximately 50% confluence. The transfection procedure was as follows: Culture medium in the 12-well plates was replaced with penicillin/streptomycin-free medium containing 2 µg/mL Lipo2000. Subsequently, the experimental groups were DDX58-WT-NC, DDX58-WT-miR, DDX58-MUT-NC, and DDX58-MUT-miR. Each sequence (Fig. 1B) was predicted using the Starbase v. 1.0 database (https://rnasysu.com/yanglab/). The DDX58-WT group received the pGL3-DDX58-WT plasmid, while the DDX58-MUT group received the pGL3-DDX58-MUT plasmid, with 1000 ng of plasmid (Hanheng Bio, China) added per well. Subsequently, 2.5 µL of NC mimics were added to each well in the NC group, and 2.5 µL of miR-21a-5p mimics were added to each well in the miR group. Both miR-21a-5p mimics and inhibitors were constructed by Ribobio (China). Finally, 200 ng of pRL-TK fluorescent control plasmid (Jike, China) was added to each well across all groups. After mixing, plates were returned to a 37 °C, 5% CO₂ incubator (Class-100, Thermo Fisher, USA) for 12 h. After 12 h of transfection, the medium was replaced with normal complete medium (12800-017, Gibco, USA). Cells were cultured for an additional 36 h before collection for the luciferase assay. Three independent transfections (biological replicates) were performed in this study, with three technical repeat wells each time.

The luciferase assay was performed according to the instructions of the Dual-Luciferase Reporter Assay System kit (E1910, Promega, USA). In brief, first prepare 1x Passive Lysis Buffer, LAR II, and Stop & GLO solution according to the manual. The cell culture medium was removed, cells were washed twice with PBS, and 100 µL of 1x Passive Lysis Buffer was added to each well to lyse the cells. Subsequently, 100 µL of LAR II solution was added, followed by 20 µL of the cell lysate. The mixture was immediately placed in a microplate reader (BioTek Synergy H1, Agilent, USA) for reading. Then, 100 µL of pre-mixed Stop&GLO solution was added, and the plate was immediately placed in the microplate reader for reading.

### Construction of the BBB injury in vitro model

Primary astrocytes (ACs) were previously isolated, and their culture followed our previous article [23]. Briefly, cells were cultured at 37 °C, 21% O₂, 5% CO₂ in DMEM medium (G4528, Servicebio, China) supplemented with 10% FBS and 1% penicillin-streptomycin. BMECs were purchased (CL-0598, Procell Life, China). CL-0598 is an immortalized mouse BMEC line, not primary BMECs. BMECs were cultured in ECM Basal Medium (37916, Sciencell, USA) supplemented with 5% FBS, 1% penicillin-streptomycin, and 1% endothelial cell growth supplement (ECGS). Subsequently, following established methods from prior studies and our previous work [23, 24], BMECs were co-cultured with ACs to establish an in vitro BBB model (Fig. S1). In brief, ACs were seeded into 6-well plates. Once ACs reached confluence, BMECs were seeded into the upper chamber of Transwell filters. After 24 h of co-culture, the BBB model (BBB) was established. The resistance (Trans Epithellal Electric Resistance, TEER) and permeability of the BBB model were measured. To simulate the endothelial cell state under Oxygen-glucose deprivation/reoxygenation (OGD/R) conditions, this study referred to the previous method [24] and first conducted contact co-culture of BCEC/AC for 24 h (Fig. S1, no need to prepare BBB). The OGD/R treatment on BBB model to generated an in vitro BBB injury model (HOBBB). The OGD/R procedure involved initially exposing cells to 2 h of hypoxia in glucose-free DMEM medium (1% O₂, 5% CO₂, 94% N₂). Subsequently, cells were cultured for 24 h in standard DMEM medium under normoxic conditions (21% O₂, 5% CO₂). The medium from the BBB-damaged in vitro model served as conditioned medium (CM) for subsequent studies. Then, 50% CM with 50% basal medium were co-incubated with BCECs, and the cells were collected for detection after OGD/R.

### Construction of DDX58 overexpression and interference plasmids and cell treatment

The overexpression plasmid was constructed using the vector PGMLV-CMV-MCS-3×Flag-PGK-Puro. The RNA interference plasmid was constructed using the vector PGMLV-CMV-MCS-EF1-ZsGreen1-T2A-Puro. The DDX58 shRNA sequences (5’→3’) are: sh-DDX58#1: ACTGGAACAGGTCGTTTATAA; sh-DDX58#2: CGGACTTCGAACACGTTTAAA; sh-DDX58#3: CAAGCATTCAGAGACTATATC.

Plasmid transfection: After replacing BMEC culture medium with penicillin/streptomycin-free medium, add 4 µL Lipo200 transfection reagent. The corresponding 2000 ng plasmid was added according to the group (OE-NC, OE-DDX58, sh-NC, sh-DDX58#1/2/3), and the cells were returned to a normoxic incubator for 24 h. After subjecting cells to the aforementioned 2 h hypoxic treatment, the medium was replaced with medium containing 50% CM, and cells were cultured in a normoxic incubator for 24 h. The group not subjected to plasmid transfection was designated as the OGD/R group.

miR-21a-5p mimic transfection: miR-21a-5p mimics were constructed by Ribobio (China). Transfection of miR-21a-5p mimics involved adding 5 µL of the mimic to the plasmid transfection procedure, with all other steps identical to plasmid transfection. Specifically, protocols differed by group: the NC group underwent mimic-NC transfection without plasmid transfection; The miR-21a-5p group did not undergo plasmid transfection but received miR-21a-5p mimic transfection; the miR + OE-NC group underwent overexpression empty vector transfection and miR-21a-5p mimic transfection; the miR + OE-DDX58 group underwent overexpression DDX58 plasmid transfection and miR-21a-5p mimic transfection.

### RT-qPCR

RT-qPCR was used to detect the RNA expression levels of DDX58 and miR-21a-5p in each group of cells. Specific methods are detailed in our previously published literature [23]. β-actin served as the internal control for calculating target RNA expression levels. Primer sequences (5’→3’) were as follows: forward: CTGGAGAAGAGCTATGAG; reverse: GATGGAATTGAATGTAGTTTC (β-actin); forward: AGCACATCATTGAAGACA; reverse: AGAGTGAAGACAGACAAC (DDX58).

Reverse transcription of miR-21a-5p was performed by stem-loop RT-PCR. Reverse Transcription was performed using the TaqMan MicroRNA Reverse Transcription Kit (4366596, Applied Biosystems, USA), following its instructions. The stem-loop RT primer and qPCR primers for miR-21a-5p were designed and synthesized by Ribobio (Guangzhou, China). U6 snRNA was used as an internal reference for miRNA (forward: 5’-CTCGCTTCGGCAGCACA-3’, reverse: 5’-AACGCTTCACGAATTTGCGT-3’). The amplification efficiency is 98.83%. The melting curve shows a single peak, with Tm approximately at 78–79 °C, and there is no non-specific peak. The relative expression levels of mRNA and miR-21a-5p were calculated by the 2^^(−ΔΔCt)^ method.

### Western blot

Western blot analysis was performed to detect the protein expression levels of DDX58 in each group of cells. The specific methodology is detailed in our previously published literature [23]. β-actin was used as the housekeeping protein for normalization of the target protein. The antibodies employed were: mouse anti-β-actin monoclonal antibody (42 kDa, 1:4000, TA-09, Zhongshan Jinqiao, China); DDX58 (101 kDa, 1:1000, 700366, Thermo, USA); TLR2 (89 kDa, 1:1000, ab209216, abcam, UK); NF-κB/p65 (65 kDa, 1:40000, 80979-1-RR, proteintech, USA); p-p65 (65 kDa, 1:10,000, 82335-1-RR, proteintech, USA); Claudin-5 (23 kDa, 1:1000, PA5-99415, Thermo, USA); Occludin (59 kDa, 1:1000, ab216327, abcam, UK); ZO-1 (195 kDa, 1:1000, ab216880, abcam, UK); MMP2 (62 kDa, 1:2000, BS-0412R, Bioss, China); MMP9 (78 kDa, 1:1000, ab283575, abcam, UK); Goat Anti Mouse IgG-HRP (M21001L, Abmart, China); Goat Anti Rabbit IgG-HRP (M21002L, Abmart, China).

### CCK-8

After removing the cell supernatant from the culture dishes, add 100 µL of basal medium to each group except the blank control. Then, add 10 µL of CCK-8 solution (CK04, Tongren Chemical, China). Place the cells back in the incubator for 2 h in the dark. Then, remove the cells and measure the OD value at 450 nm using a microplate reader to calculate cellular metabolic activity.

### Immunofluorescence staining

Cells on coverslips were fixed with 4% paraformaldehyde at 4 °C. After 30 min, coverslips were blocked with 5% sheep serum solution. Excess blocking solution was discarded, and the primary antibody in 2% sheep serum solution was incubated overnight at 4 °C. After rewarming, the secondary antibody was added and incubated at 37 °C for 1 h. Subsequently, mount each slide with 50 µL of anti-fade mountant (containing DAPI) and observe under an upright fluorescence microscope (BX53, Olympus, Japan) for imaging. Objective lens: 40×/ 0.75NA. Each sample randomly selected from 6 vision (random field selection), to avoid the edge and bubble areas. For background subtraction using ImageJ software (rolling ball radius 50 pixels) and mean fluorescence intensity (mean gray value) quantitatively. Fluorescence intensity normalization to every field of vision DAPI positive number of nuclei. Antibodies used: DDX58 (1:300, 700366, Thermo, USA); ZO-1 (1:200, ab216880, abcam, UK); Affinity-purified antibody 488-labeled goat Anti-rabbit IgG (H + L) (1:1000, 5230 − 0385, KPL, China); Affinity-purified antibody Cy3-labeled goat anti-rabbit IgG (H + L) (1:1000, 072-01-15-06, KPL, China).

### Construction and treatment of animal models

As previously described, the construction of the mouse model of hemorrhagic transformation following middle cerebral artery occlusion (MCAO-HT) and treatment [17], the in vivo samples used in this study were archived specimens from previous articles and were subjected to secondary molecular analysis. C57BL/6J mice (18–22 g) were anesthetized with 0.3% sodium pentobarbital, followed by ligation of the distal common carotid artery (CCA) using hemostatic forceps. A live knot was tied at the distal end of the pre-external carotid artery (PECA) and at the junction of the CCA and PECA. A silicone rubber plug was inserted between the two knots, and the live knots were tightened. After 5 h of continuous anesthesia with sevoflurane gas, the silicone plug was removed and the neck skin sutured. The sham group (Sham) mice underwent arterial exposure only. After 24 h, model group mice (I/R, *n* = 60) and sham group mice (Sham, *n* = 15) were obtained. BMSC exosomes (BMSC-exo) were extracted using the Ribo Exosome Isolation Kit (C10130, Ribobio, China) and adjusted to a concentration of 10^8^ exosomes/µL. The I/R model mice were divided into three groups: the BMSC-exo treatment group (I/R + exo, *n* = 30), the I/R + exo+inhibitor miR group (*n* = 15), and the I/R + exo+mimic miR group (*n* = 15) received tail vein injections of 100 µL BMSC-exo, 100 µL of BMSC-exo transduced with miR-21a-5p inhibitor and 100 µL of miR-21a-5p mimic lentivirus (5 × 10^7^ TU). One week later, mice were anesthetized by carbon dioxide asphyxiation and euthanized by cervical dislocation. Similarly, the in vitro BBB injury model treated with BMSC-exo, miR-21a-5p inhibitor, or mimic yielded three in vitro model treatment groups (*n* = 3): HOBBB + exo, HOBBB + exo+inhibitor miR, and HOBBB + exo+mimic miR. All animal experiments complied with ARRIVE guidelines and were approved by the Kunming Medical University Ethics Committee (kmmu20241922). During the experiment, result evaluation and data analysis, the testers were unaware of the group allocation.

### Detection of MMPs enzyme activity and cell resistance and permeability

The MMP activity detection (fluorescence method - green) kit (ab112146, Abcam, UK) was used, and the MMP activity detection was carried out strictly in accordance with the instructions. The TEER of the non-contact in vitro co-culture model (Fig. S1) was detected by a resistance meter. Fluorescent yellow permeability detection, in simple terms, involves adding 100 µL of Hanks’ Balanced Salt Solution containing a final concentration of 100 µg/mL of fluorescent yellow to the top chamber of the Transwell chamber, incubating at 37 °C for 4 h, and then collecting the liquid in the basal side chamber. The absorbance (excitation wavelength 427 nm, emission wavelength 536 nm) was measured by a fluorescence spectrophotometer, and the concentration of fluorescent yellow was calculated.

### Statistical analysis

Data were expressed as the mean ± SD from three independent samples per group. Normality of distribution was verified using the Shapiro-Wilk test. For multiple group comparisons, one-way ANOVA was performed, followed by Tukey HSD post hoc multiple comparisons. Differences were considered statistically significant at *p* < 0.05. *n* = 3 (cell experiment): refers to three independent cultures/transfections. Three technical replicate wells or lanes were set up inside each independent experiment. In animal experiments, n refers to the number of individual animals.

## Results

### miR-21a-5p targets DDX58

Our previous research identified miR-21a-5p as the most significantly differentially expressed microRNA in the ipsilateral tissue of ischemic brain injury, as validated by RT-qPCR [17]. Bioinformatics analysis predicted DDX58 as a potential target of miR-21a-5p (Fig. 1A). This interaction was further validated through dual luciferase reporter assays, which demonstrated that miR-21a-5p specifically suppressed luciferase activity in wild-type DDX58 sequences, but had no significant effect on mutant sequences, confirming direct binding (Fig. 1B). Expression analysis of DDX58 in the sham, ischemia/reperfusion (I/R), and BMSC-exo-treated groups revealed a significant upregulation of DDX58 expression in I/R mice, which was markedly reduced following BMSC-exo treatment (Fig. 1C). In an in vitro BBB injury model induced by hypoxia, BMSC-exo treatment significantly decreased both mRNA and protein levels of DDX58. In contrast, inhibition of miR-21a-5p expression led to an increase in DDX58 expression, whereas miR-21a-5p overexpression resulted in the opposite effect (Fig. 1D-F). Similarly, in vivo I/R-induced BBB injury models showed that BMSC-exo treatment reduced DDX58 expression, while miR-21a-5p inhibition significantly elevated DDX58 expression, with miR-21a-5p mimetics exerting the opposite regulatory pattern (Fig. 1G-I). These findings suggest that BMSC-exo transport miR-21a-5p into BMECs, targeting and regulating DDX58 in vitro to modulate cellular phenotypes. While in vivo data support an association between DDX58 expression and BBB injury severity, the causal role of DDX58 in vivo remains to be determined. In vitro, DDX58 serves as a key intermediary target gene of miR-21a-5p in the amelioration of BBB injury.

### DDX58 promots BBB damage

**Fig. 2:**
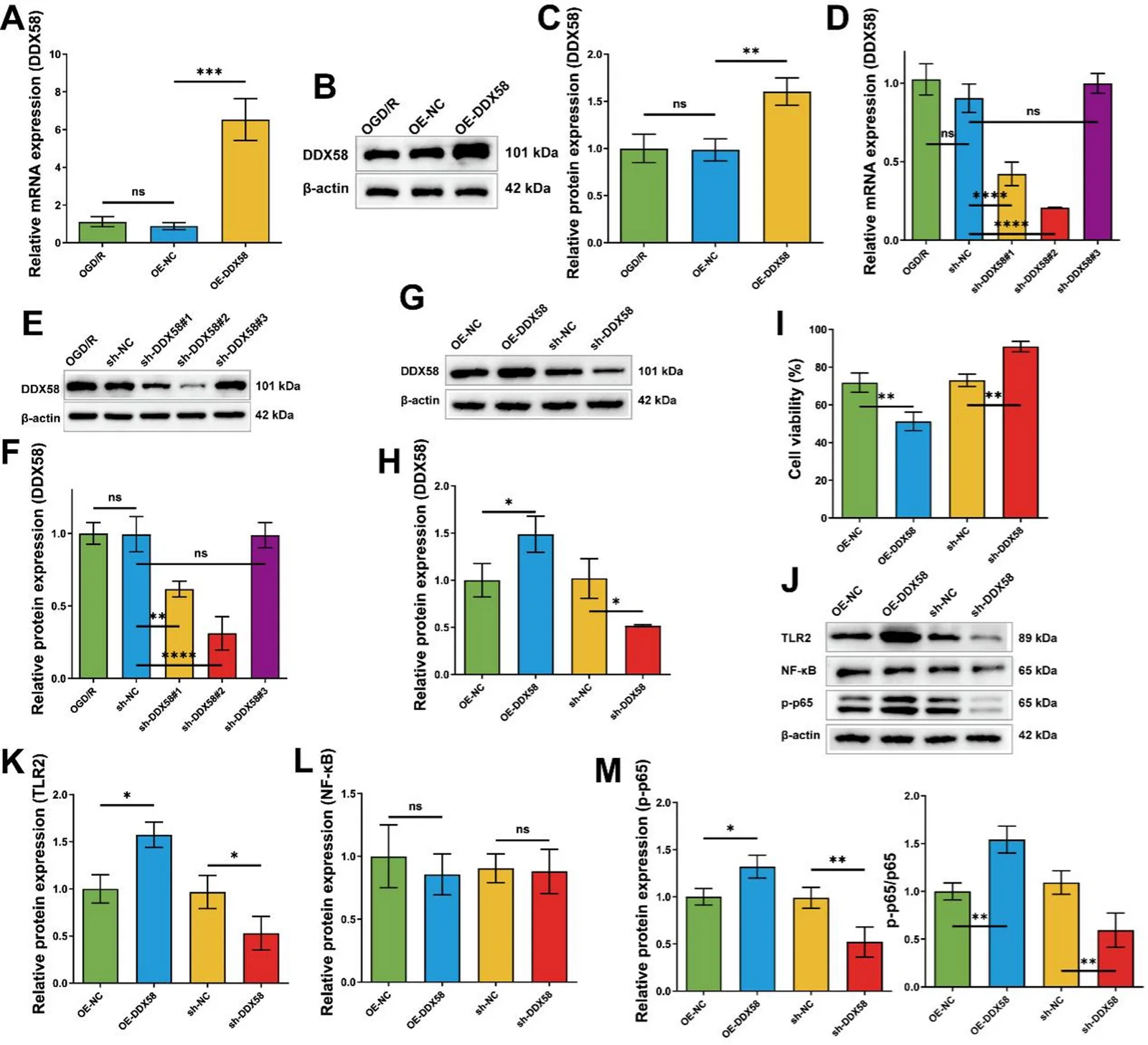
DDX58 manipulation is associated with changes in TLR2/NF-κB-related markers and inhibits BMEC viability. **A-C**: The mRNA (A) and protein (B-C) expression of DDX58 in BMECs after transfection with the overexpression plasmid was detected by RT-qPCR and western blot. **D-F**: The mRNA (D) and protein (E-F) expression of DDX58 in BMECs after transfection with an interfering plasmid were detected by RT-qPCR and Western blot. **G-H**: The protein expression of DDX58 in each group of the BBB injury cell model was detected by western blot. **I**: Cellular metabolic activity was detected by CCK-8 assay. **J-M**: The protein expression of TLR2 (K), NF-κB (L), and p-p65 (M) were detected by western blot. ns indicates no significant difference between two groups; *p < 0.05; ** *p* < 0.01; ***p < 0.001; ****p < 0.0001

**Fig. 3:**
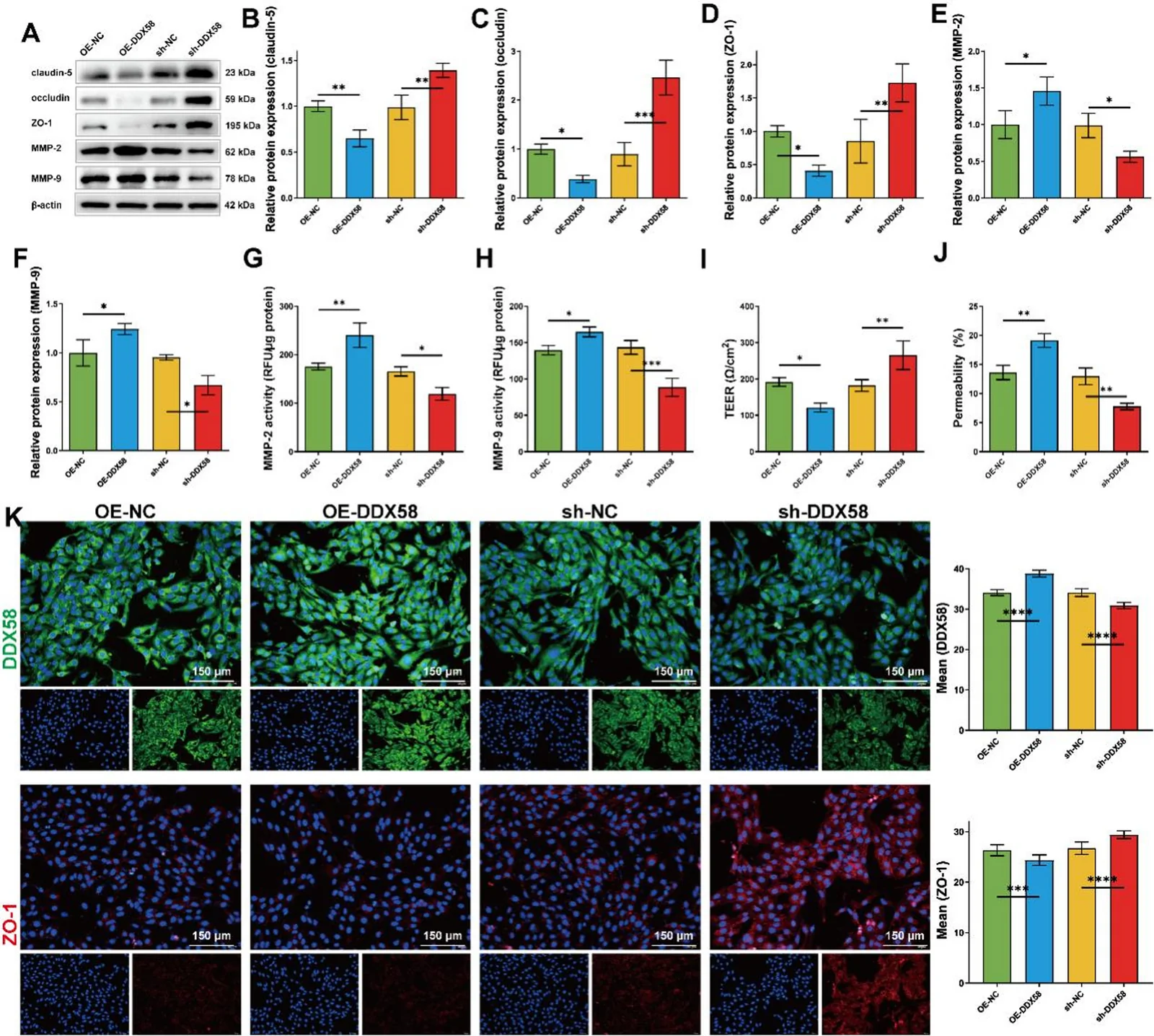
DDX58 promotes BBB damage. **A–F**: Western blot analysis (A) for blood-brain barrier tight junction proteins claudin-5 (B), occludin (C), ZO-1 (D), and BBB injury-associated proteins MMP-2 (E) and MMP-9 (F). **G-H**: The enzymatic activities of MMP-2 (G) and MMP-9 (H) were detected by the kit. **I-J**: Detection of TEER and permeability of cells. **K**: The expression of DDX58 and ZO-1 was detected by immunofluorescence staining; Scale bar: 150 μm. ns indicates no significant difference between two groups; *p < 0.05; **p < 0.01; ***p < 0.001; ****p < 0.0001

To further explore how miR-21a-5p-mediated targeting of DDX58 affects BMEC behavior and contributes to BBB injury, we performed DDX58 interference and overexpression experiments in BMECs (Fig. 2A-H). The sh-DDX58#2 with the highest knocking efficiency was used for the follow-up study (Fig. 2D-F). Our results demonstrated that overexpression of DDX58 significantly impaired BMEC metabolic activity, whereas DDX58 silencing enhanced BMEC metabolic activity (Fig. 2I). Mechanistically, although DDX58 did not significantly affect total NF-κB protein levels, overexpression of DDX58 led to increased expression of TLR2 and phosphorylated p65 (p-p65), while DDX58 knockdown inhibited these effects (Fig. 2J-M). Additionally, tight junction proteins claudin-5, occludin, and ZO-1 were significantly downregulated by DDX58 overexpression, while DDX58 knockdown reversed this effect (Fig. 3A-D). Overexpression of DDX58 also significantly increased MMP-2/9 expression, whereas knockdown of DDX58 inhibited MMP-2/9 expression (Fig. 3A, E-F) and enzymatic activity of MMP-2/9 (Fig. 3G-H). In addition, overexpression of DDX58 significantly reduce the TEER of cells and improve their permeability (Fig. 3I-J). Immunofluorescence analysis of DDX58 and ZO-1 expression corroborated these findings (Fig. 3K). These results indicate that DDX58 manipulation is associated with changes in TLR2/NF-κB-related markers, increased MMP-2/9 expression, and reduced tight junction protein expression, suggesting a promoting role in BBB damage.

### miR-21a-5p targets DDX58 to improve BBB injury

**Fig. 4:**
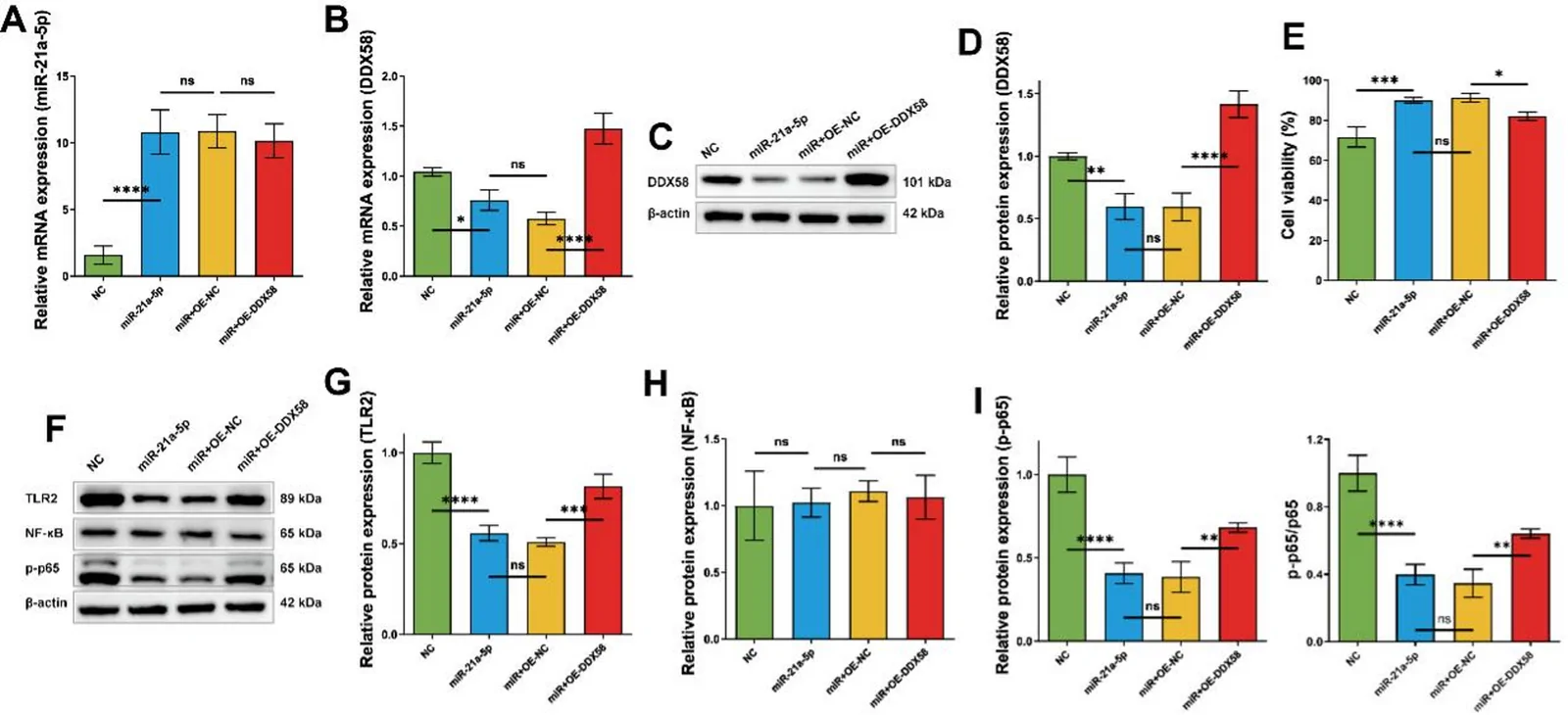
DDX58 reverses the effect of miR-21a-5p, with associated changes in TLR2/NF-κB-related markers, and inhibits BMEC viability. **A**: The expression of miR-21a-5p was detected by RT-qPCR. **B-D**: The expression of DDX58 mRNA (B) and protein (C-D) was detected by RT-qPCR and western blot. **E**: Cellular metabolic activity was detected by CCK-8 assay. **F-I**: The expression of TLR2 (G), NF-κB (H), and p-p65 (I) was detected by western blot. ns indicates no significant difference between two groups; *p < 0.05; **p < 0.01; ***p < 0.001; ****p < 0.0001

**Fig. 5:**
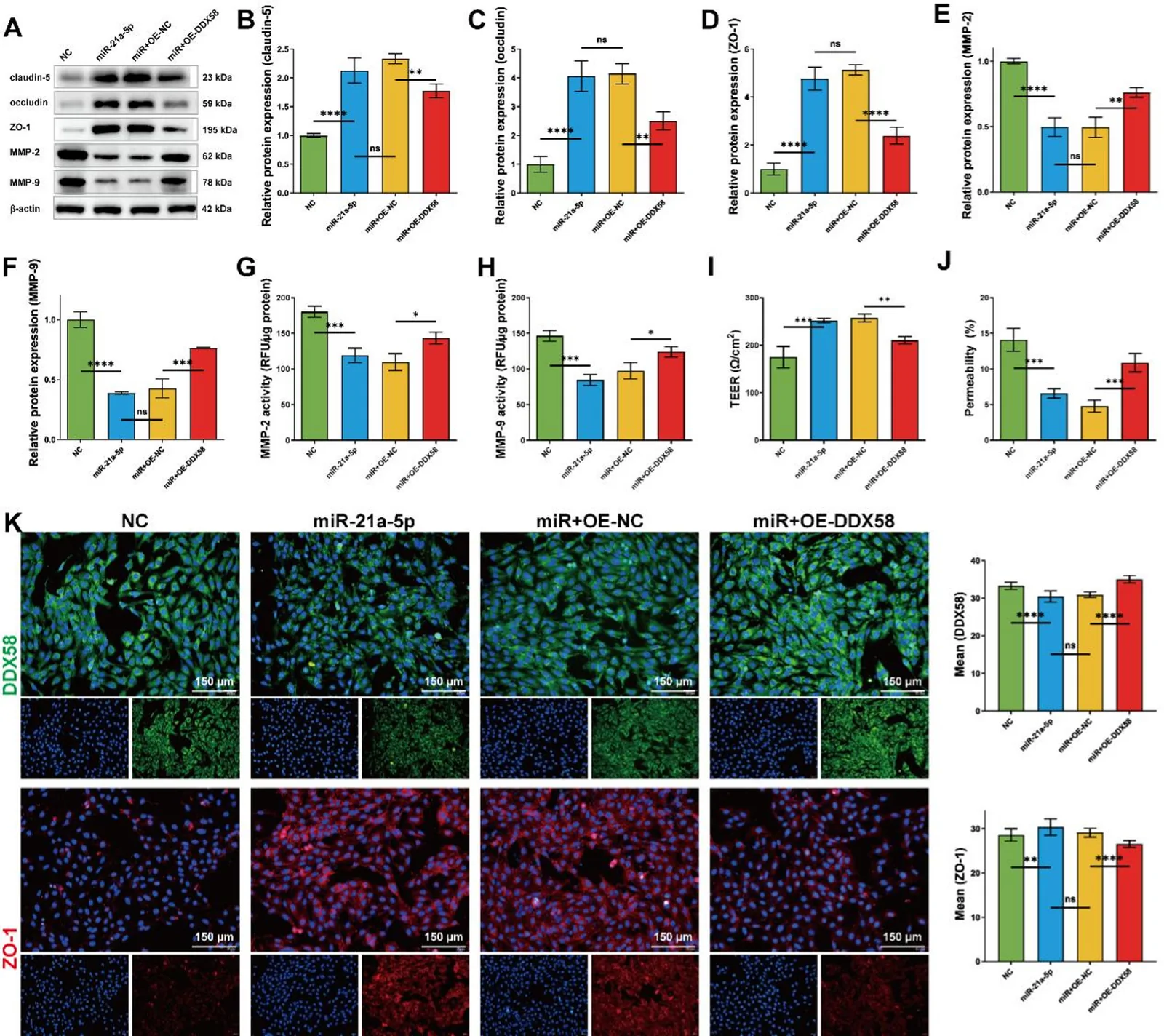
DDX58 reverses the effects of miR-21a-5p and promotes BBB damage. **A-F**: Western blot analysis (A) detected the expression of BBB tight junction proteins claudin-5 (B), occludin (C), ZO-1 (D), and BBB injury-related proteins MMP-2 (E) and MMP-9 (F). G-H: The enzymatic activities of MMP-2 (G) and MMP-9 (H) were detected by the kit. I-J: Detection of TEER and permeability of cells. K: The expression of DDX58 and ZO-1 was detected by immunofluorescence staining; Scale bar: 150 μm. ns indicates no significant difference between two groups; **p < 0.01; ***p < 0.001; ****p < 0.0001

To validate the mechanism by which miR-21a-5p targets DDX58 and improves BBB injury, rescue experiments were performed. The results confirmed that DDX58 is downstream of miR-21a-5p, with DDX58 overexpression not significantly affecting miR-21a-5p RNA levels (Fig. 4A). In the in vitro BBB injury model, treatment with miR-21a-5p significantly suppressed DDX58 expression and promoted BMEC metabolic activity, while DDX58 overexpression reversed these effects (Fig. 4B-E). Moreover, miR-21a-5p treatment significantly inhibited the TLR2/NF-κB pathway, whereas simultaneous overexpression of DDX58 during miR-21a-5p treatment activated the TLR2/NF-κB pathway (Fig. 4F-I). Further mechanistic studies revealed that miR-21a-5p treatment upregulated the expression of tight junction proteins claudin-5, occludin, and ZO-1, while DDX58 overexpression suppressed this effect (Fig. 5A-D). Additionally, miR-21a-5p treatment significantly decreased MMP-2/9 expression and enzymatic activity, while DDX58 overexpression led to a significant increase in MMP-2/9 expression and enzymatic activity (Fig. 5A, E-H). In addition, miR-21a-5p treatment significantly increased TEER and reduced cell permeability, while overexpression of DDX58 significantly reduce the TEER of cells and improve their permeability (Fig. 3I-J). Immunofluorescence analysis of DDX58 and ZO-1 expression confirmed the protein expression results (Fig. 5K). These findings indicate that miR-21a-5p targets DDX58, which is associated with altered expression of TLR2/NF-κB-related markers, promotion of tight junction protein expression, and inhibition of BBB degradation proteins, thereby exerting a protective effect on the BBB.

## Discussion

This study further investigates the molecular mechanisms through which miR-21a-5p mitigates blood-brain barrier damage. Our findings demonstrate that miR-21a-5p directly targets and suppresses DDX58. Inhibition of DDX58 expression is associated with reduced levels of TLR2/NF-κB-related markers, accompanied by an increase in tight junction proteins, including claudin-5, occludin, and ZO-1, while concurrently reducing the expression of MMP-2/9, which are associated with BBB structural degradation. These changes collectively contribute to the amelioration of BBB damage.

Previous work by our group established that exosomes containing miR-21a-5p exert protective effects on the BBB via the TLR2/NF-κB pathway. However, the specific molecular targets of miR-21a-5p remained unidentified [17]. This study identifies DDX58 as a key molecular target through bioinformatics and dual luciferase assays. The results of this study demonstrate that DDX58 is abnormally overexpressed in both in vivo and in vitro models of BBB injury, establishing a direct association between DDX58 and BBB damage.

Several studies have highlighted the role of miRNAs in modulating immune responses and inflammation by targeting DDX58, such as miR-709 [25], miR-139 [26], miR-526a [27], miR-223-3p [28], and miR-136 [29]. Moreover, DDX58 is known to participate in the amplification of NF-κB signaling [30], further supporting its involvement in inflammation-induced BBB damage. Despite these insights, the specific mechanisms by which DDX58 influences BBB endothelial cells, particularly through inflammation-related pathways, remain incompletely understood. Building on prior findings from our group [17], we demonstrate that DDX58 manipulation is associated with changes in TLR2/NF-κB-related markers, increased matrix metalloproteinase expression, and degradation of tight junction proteins, contributing to BBB injury. Specifically, DDX58 overexpression is associated with increased TLR2 expression and p65 phosphorylation, which parallels NF-κB activation patterns reported in previous studies [31]. Furthermore, overexpression of DDX58 in miR-21a-5p-treated in vitro BBB injury models reversed the protective effects of miR-21a-5p, providing in vitro validation of miR-21a-5p’s mechanism of action through targeting DDX58. It is important to note that while DDX58 has been reported to participate in the amplification of NF-κB signaling, the relationship between DDX58 and TLR2 is not a simple linear upstream-downstream hierarchy. Rather, previous studies have demonstrated that DDX58 and TLR2 form a complex bidirectional regulatory crosstalk network [32]. In the present study, we observed that DDX58 overexpression significantly upregulated TLR2 expression, whereas DDX58 knockdown inhibited TLR2, revealing a previously uncharacterized non-canonical regulatory association. In the OGD/R inflammatory microenvironment, DDX58 may indirectly upregulate TLR2 transcription through NF-κB positive feedback loops [33], or alternatively, achieve pathway crosstalk via inflammasome/cytokine-mediated paracrine effects [32]. These possibilities warrant further mechanistic investigation to delineate the precise mode of DDX58-TLR2 interaction in BBB endothelial cells under ischemic stress.

While this study elucidates the protective role of miR-21a-5p in BBB repair by targeting DDX58, several limitations should be considered. First, although the effects of miR-21a-5p on BBB injury were validated both in vitro and in vivo, the specific mechanism by which miR-21a-5p targets DDX58 was primarily explored in cellular models. It is important to note that direct in vivo manipulation of DDX58 was not performed. Therefore, the causal mediation of BBB protection by DDX58 in vivo cannot be concluded from the present data. The in vivo validation was limited to expression analysis, and further in vivo studies are needed to enhance the robustness of these findings. Second, although we have confirmed the involvement of the TLR2/NF-κB pathway in miR-21a-5p-DDX58-mediated BBB repair, other potential pathways remain unexplored. Future research should investigate whether additional signaling pathways contribute to this process. Nonetheless, it is clear that DDX58 plays a central role in the miR-21a-5p-mediated protection of the BBB. the uptake process of BMSC-exo was not independently and repeatedly verified in this study. Additionally, the relevant conclusions rely on the characterization data from previous studies [17] and the functional consistency inference of this study. This study lays the groundwork for developing targeted therapeutic strategies, including the use of precision delivery systems featuring a triple modification of “miRNA-exosome-targeted peptides.” Multi-center, multi-species studies will be crucial for advancing the clinical translation of the miR-21a-5p-DDX58-TLR2/NF-κB regulatory axis.

## Conclusion

Our in vitro findings demonstrate that miR-21a-5p exerts a protective effect against BBB injury by targeting and inhibiting DDX58. This action blocks the TLR2/NF-κB signaling pathway, leading to the promotion of tight junction protein expression and suppression of MMP-2/9 activity, thus mitigating BBB damage. In vivo data support an association between miR-21a-5p-mediated DDX58 downregulation and attenuated BBB damage.

## Supplementary Information

Below is the link to the electronic supplementary material.

Supplementary Material 1

## Data Availability

Data will be made available by the corresponding author on request.
