# Supplementary material for: miR-21a-5p targets DDX58 to suppress TLR2/NF-κB signaling and protect the blood–brain barrier after injury
Source: Mol Biol Rep. 2026 Oct 25;53(1):1812. doi: 10.1007/s11033-026-13010-3 (PMC13696569; doi:10.1007/s11033-026-13010-3)
Supplement: Supplementary file 1 [file 11033_2026_13010_MOESM1_ESM.docx]

**Figure S1:** The in vitro experimental workflow.
